# Feasibility of an App-Assisted and Home-Based Video Version of the Timed Up and Go Test for Patients with Parkinson Disease: vTUG

**DOI:** 10.3390/jcm14113769

**Published:** 2025-05-28

**Authors:** Marcus Grobe-Einsler, Anna Gerdes, Tim Feige, Vivian Maas, Clare Matthews, Alejandro Mendoza García, Laia Comas Fages, Elin Haf Davies, Thomas Klockgether, Björn H. Falkenburger

**Affiliations:** 1 German Center for Neurodegenerative Diseases (DZNE), 53127 Bonn, Germany; marcus.grobe-einsler@ukbonn.de (M.G.-E.);; 2 Center for Neurology, Department of Parkinson’s Disease, Sleep and Movement Disorders, University Hospital Bonn, 53127 Bonn, Germany; 3 Department of Neurology, Faculty of Medicine, TUD Dresden University of Technology, 01307 Dresden, Germany; anna.gerdes@ukdd.de (A.G.);; 4 German Center for Neurodegenerative Diseases (DZNE), 01307 Dresden, Germany; 5 Aparito Ltd., a wholly owned subsidiary of Eli Lilly and Company, Wrexham LL13 7YP, UK

**Keywords:** digital biomarkers, motor assessments, home monitoring

## Abstract

**Background**: Parkinson Disease (PD) is a progressive neurodegenerative disorder. Current therapeutic trials investigate treatments that can potentially modify the disease course. Testing their efficiency requires outcome assessments that are relevant to patients’ daily lives, which include gait and balance. Home-based examinations may enhance patient compliance and, in addition, produce more reliable results by assessing patients more regularly in their familiar surroundings. **Objective**: The objective of this pilot study was to assess the feasibility of a home-based outcome assessment designed to video record the Timed up and Go (vTUG) test via a study-specific smartphone app for patients with PD. **Methods**: 28 patients were recruited and asked to perform at home each week a set of three consecutive vTUG tests, over a period of 12 weeks using an app. The videos were subjected to a manual review to ascertain the durations of the individual vTUG phases, as well as to identify any errors or deviations in the setup that might have influenced the result. To evaluate the usability and user-friendliness of the vTUG and app, the System Usability Scale (SUS) and User Experience Questionnaire (UEQ) were administered to patients at the study end. **Results**: 19 patients completed the 12-week study, 17 of which recorded 10 videos or more. A total of 706 vTUGs with complete timings were recorded. Random Forest Regression yielded “time to walk up” as the most important segment of the vTUG for predicting the total time. Variance of vTUG total time was significantly higher between weeks than it was between the three consecutive vTUGs at one time point [F(254,23) = 6.50, *p* < 0.001]. The correlation between vTUG total time and UPDRS III total score was weak (r = 0.24). The correlation between vTUG and a derived gait subscore (UPDRS III items 9–13) was moderate (r = 0.59). A linear mixed-effects model revealed a significant effect of patient-reported motion status on vTUG total time. Including additional variables such as UPDRS III gait subscore, footwear and chairs used further improved the model fit. **Conclusions**: Assessment of gait and balance by home-based vTUG is feasible. Factors influencing the read-out were identified and could be better controlled for future use and longitudinal trials.

## 1. Introduction

Parkinson disease (PD) has the fastest growing worldwide prevalence of all neurologic diseases [1]. Motor symptoms are a major driver of reduced quality of life and are caused by degeneration of dopaminergic neurons in the substantia nigra. Several factors contribute to the degeneration of dopaminergic neurons, including protein aggregation, aberrant proteostasis, altered energy homeostatis, inflammation and alterations in neurotransmitter systems such as the orexinergic system contribute to PD pathogenesis [2,3,4,5]. Current symptomatic treatment strategies focus on elevation of dopamine levels in the brain. In addition, first disease-modifying treatments are under investigation. These therapies aim to delay the disease progression. Obstacles for conducting such clinical trials include the need for reliable outcome measures that are sensitive to subtle changes of symptom severity and relevant to patients’ daily lives.

Several authors have argued that patients with PD (PwPD) should be tested in their home environment whenever possible [6,7,8,9]. Indeed, exhaustion during long trial visits can influence the results of clinical assessments and frequent travels to the study site represent a burden for patients with PD, in particular for patients in advanced stages of the disease. Home assessments are increasingly facilitated by the use of Digital Health Technologies. They can be applied in a remote setting for longitudinal monitoring and potentially more sensitive to subtle changes of motor function compared to conventional clinical scales [10]. The benefits of Digital Health Technologies in monitoring PwPD are increasingly recognized, with the coronavirus pandemic acting as an additional catalyst for their application as remote assessments [11,12].

The “Timed Up and Go”-Test (TUG) is well accepted in the field and easy to apply [7,13]. In this test, the patient is asked to stand up from a chair, walk three meters in a straight line, turn around, walk back, and sit back down. The objective assessment is made by measuring the time to complete the task. It was originally designed to evaluate dynamic balance, functional mobility and risk of falls in geriatric patients [14,15], but was later demonstrated to be a valuable and efficient method for evaluation of mobility in PwPD [16,17] with a high test-retest reliability [18,19]. Yet, the TUG does not report all aspects of motor impairment in PD. Several approaches to digitize the TUG with sensors have been introduced, which are summarized under the term instrumented TUG (iTUG) [20]. The sensors allow for accurate subdivision of the TUG phases by postural transitions, and introduce qualitative gait analysis, which ultimately leads to improved discriminatory properties in early disease stages [20,21]. Building on the recent advances in smartphone camera technology, we developed and validated a self-applied, video recorded, assisted remote TUG for home application, named vTUG, via a smartphone app.

## 2. Materials and Methods

### 2.1. Recruitment

In total, 33 PwPD were recruited in the German Center for Neurodegenerative Diseases at the Bonn and Dresden sites between January and December 2023. Five patients participated in the in-clinic feasibility assessment; 28 patients participated in the home-based longitudinal part. Inclusion criteria were (i) individuals aged 18 and above who met the diagnostic criteria for PD as stipulated by the Movement Disorder Society [22]; (ii) Hoehn and Yahr Stages 1 to 4, (iii) the ownership of an Android or iOS Smartphone with internet access, (iv) ability to comply with study protocol without the risk of falling as judged by the investigator. Exclusion criteria were severe comorbidities that could interfere with assessments such as dementia, risk of falling or severe psychiatric disease.

### 2.2. Ethics Approval

This study was conducted in accordance with the Declaration of Helsinki. The study was approved by the local ethics committees: Ethikkommission an der Medizinischen Fakultät Bonn. Reference 212/19, date 29 October 2021 and Ethikkommission der Technischen Universität Dresden. Reference BO-EK-149032021_3, date 27 April 2024). Written informed consent was obtained from all patients before study participation.

### 2.3. App Implementation

The vTUG module was implemented in an e-health app (Atom5™ by Aparito, Wrexham, UK), with compatibility for Android and iOS to enable patients to use their own phone or tablet. After initialisation via a unique patient identifier QR code, the activated module contained instructional videos and text on how to perform the vTUG, available in both English and German. The actual performance was recorded within the app and uploaded for central review and assessment. Before completing the vTUG assessment, patients were asked to respond to questionnaires from two categories: The first was a “health thermometer” (numeric value from 0–100) as a measure for general health. The second consisted of a self-evaluation of motion (ON or OFF), the time since last dopaminergic medication and changes in medication since the last assessment. At the end of the study, patients were asked to respond to the User Experience Questionnaire (UEQ) [23] and the System Usability Scale (SUS) [24]. The SUS is a widely used Likert-Scale and ten-item questionnaire with five possible response options. The SUS is more focused on evaluating the usability of a system, while the UEQ has a broader scope, encompassing the overall user experience. The UEQ assesses Attractiveness, Perspicuity, Efficiency, Dependability, Stimulation, and Novelty of a technical system. Patients received push messages via the app as reminders for upcoming assessments for the duration of the study.

### 2.4. Study Design and Setup

Clinical information was collected during the baseline visit in the study center and included age, sex, Hoehn and Yahr stages, and disease onset. The MDS-UPDRS III scores were obtained in Bonn from a longitudinal cohort study in which the participants took part, and in Dresden from the medical records of the Department of Neurology at the University Hospital. Each score corresponded to the day of recruitment. A UPDRS III gait subscore was calculated as the sum of gait-associated items 9–13 of the MDS-UPDRS III.

The first stage of the project was an in-clinic usability study to assess if patients were able to follow the in-app instructions provided to perform the vTUG task independently. Five patients were asked to navigate the app by themselves, under the surveillance of a clinician. The clinician noted difficulties that the patients experienced and whether they required help. Feedback regarding user friendliness from this phase was subsequently implemented in the app. These changes included bigger font size, easier navigation through the app, optimization of the instructions and translations.

The second stage consisted of a longitudinal home monitoring. The investigations proceeded as follows: The app installation and initialisation process and first assessment were carried out at the study site under the supervision of an investigator, who provided advice if requested. To start the assessment, patients were asked to watch the video instructions, respond to the first questionnaires and prepare the set-up as follows: A floor-mark was placed at 3 m distance from a chair. A provided tripod with the mounted smartphone was placed another 2 m along the same line. The chair, floor mark and tripod were to be aligned without any obstacles in between. The height and position of the smartphone was adjusted to capture the standing patient on the 3 m floor mark from head to toe. The patient was then asked to sit down in the chair to start the assessment. The recording was initiated either beforehand by the patient, via voice command when the patient was already sitting in the chair, or via a second person. During the vTUG assessments, patients stood up (without assistance of the arms, if possible), walked 3 m at normal walking speed, turned around, walked back, and sat back down again. This sequence was repeated three times and each recorded on video. The first session was performed in clinic under supervision of an investigator; the consecutive weekly questionnaires and recordings were performed independently by patients at their homes, once a week for 12 weeks, again in triplicates. To avoid consecutive erroneous performances, the first home recording was centrally reviewed by an investigator in the same week and patients were contacted to correct performance in the following recording, if necessary. Additionally, patients were able to contact investigators from their local study site if they had questions.

### 2.5. Data Analysis

All videos underwent manual quality control for completeness of the recording and correct framing of the person. The five stages making up the TUG (stand, walk up, turn 180°, walk back and turn to sit) were timed using definitions developed to identify the start and end of each stage. The duration of pauses between stages and the total time were also recorded. For each patient, it was also noted how many times and in which videos they were in a different location, wearing different footwear or using a different chair from the original setup.

Participants performed the vTUG three times consecutively at each time point, resulting in three individual vTUG measurements per session. The mean of these three trials was calculated to represent the participant’s performance at that time point.

#### 2.5.1. Relevance of vTUG Stages on Total Time

To assess the relevance of the five TUG stages described above on the total TUG time, we calculated both Pearson correlation coefficients and performed a Random Forest regression analysis. For the Random Forest, we conducted a grid search to identify the optimal hyperparameters for the minimum number of samples per leaf and the number of trees in the forest.

#### 2.5.2. Correlation with MDS-UPDRS III

To evaluate the suitability of the home-based vTUG for measuring motion and particularly gait impairments in people with Parkinson’s disease, we calculated Pearson correlation coefficients and performed individual linear regressions of vTUG total time on the MDS-UPDRS III total score and MDS-UPDRS III gait score.

#### 2.5.3. Variance

To assess the vTUG’s ability to detect changes in walking performance, we calculated the within-session variance among the three vTUG trials conducted at each time point, as well as the variance between these mean values over the 12-week study period. To evaluate whether the variances differed significantly within and between time points, we performed an F-test, calculating the F-statistic and corresponding *p*-value.

#### 2.5.4. Influences on Total Time

To identify relevant variables for predicting the dependent variable vTUG total time we estimated a mixed linear model using the mixedlm function from the statsmodels package (v. 0.14.2) in Python with the UPDRSIII total, UPDRSIII gait subscore, patients’ perceived motor status, age, disease duration as well as shoes worn, chairs used and locations filmed at as predictor variables. We included random intercepts for each participant to account for the repeated measures design, allowing us to control for individual variability in baseline performance. We employed a stepwise model-building procedure, sequentially adding the predictor variables in the order listed above. At each step, we compared the Akaike Information Criterion (AIC) and Bayesian Information Criterion (BIC) and conducted a Likelihood Ratio Test (LRT) between the reduced model (excluding the new variable) and the full model (including the new variable) to determine whether to retain the variable in the model. In order to calculate AIC, BIC and perform LRTs we estimated all models using maximum likelihood.

To assess the final model fit, we calculated the marginal and conditional R^2^ values using Nakagawa and Schielzeth’s formula [25].

#### 2.5.5. Usability

To assess the usability of the vTUG we applied the System Usability Score (SUS) [24] and the User Experience Questionnaire (UEQ) [23].

The SUS provides a subjective assessment of usability from the patient’s perspective through ten Likert scale questions. For odd-numbered questions, participants rate from 1 (strongly disagree) to 5 (strongly agree), while even-numbered questions are rated inversely from 5 to 1. The SUS score is calculated by adjusting the responses—subtracting 1 from each odd-numbered question and subtracting the response from 5 for each even-numbered question. These adjusted scores are summed and multiplied by 2.5, resulting in a total score ranging from 0 to 100. Higher scores indicate better perceived usability, with scores above 68 considered above average and those exceeding 80 considered excellent [26].

The UEQ is a standardized questionnaire designed to assess the user experience of products, systems, or services. It consists of 26 items that measure six key dimensions: Attractiveness, Perspicuity (clarity), Efficiency, Dependability, Stimulation, and Novelty. Participants rate each item on a seven-point scale ranging from −3 (extremely bad) to +3 (extremely good). Instead of generating an overall score, mean values for each domain are analyzed, with values greater than 0.8 considered a positive evaluation [27].

## 3. Results

In total, 28 patients were included in this study. Three participants dropped out after the in-clinic assessment due to personal reasons that were not linked to the burden of the study. The clinical and demographic data for the remaining 25 patients are summarized in Table 1.

### 3.1. Video Summary

A total of 273 videos were recorded, of which 262 passed quality control and were included in the analysis. Four videos were unavailable due to upload failures, and seven were excluded because the camera was partially covered or did not capture a vTUG at all.

**Figure 1:**
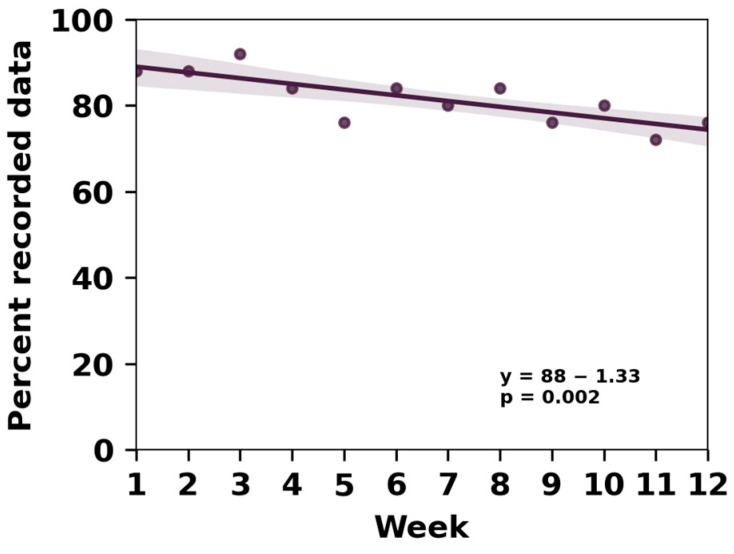
Despite a small attrition rate, data availability remained quite high throughout the study duration.

Eight patients completed the task every week, submitting 12 videos each that included 36 vTUGs. Eleven additional patients also completed the 12-week period but missed some weekly video recordings due to unspecified issues (Figure 1). Over the course of the study, we observed only a small attrition rate, with the percentage of available data decreasing from 88% in week 1 to 76% in week 12. Linear regression analysis estimated a 1.3% decrease in available data per week.

**Figure 2:**
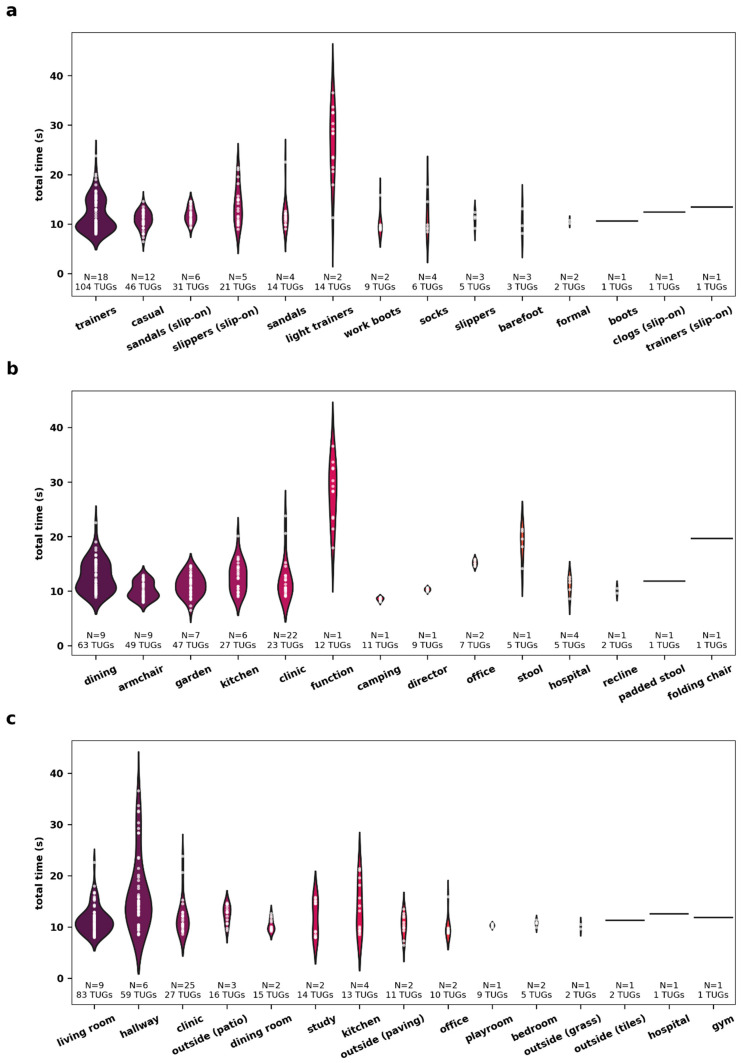
Different shoes, chairs, and locations introduce notable variability in vTUG total time. Violin plots display the distribution of vTUG total time by the shoes worn (**a**), chairs used (**b**), and locations filmed at (**c**). The number of patients per category (N) and the total number of TUGs performed are indicated for each category.

The 262 videos contained 784 vTUG assessments (two videos contained only two instead of the standard three vTUGs). Out of these, 706 covered the entire task; timing was incomplete in 78 vTUGs because the recordings failed to capture the beginning of the first TUG test. In nine videos, the participants’ feet were out of frame at the 3-m mark, mostly due to walking past the floor mark or improper setup. In 12 videos—seven of which were recorded by a single patient—the participants’ shoulders were out of frame during the turn at the 3-m mark. Most patients consistently used the same chair at home, with only a few changing it once or twice. The most frequent changes were observed in footwear: only nine patients consistently used the same pair of shoes, while the others changed their shoes up to seven times. Additionally, the majority of patients recorded each TUG at the same location, eight patients change locations once or twice. The variability in vTUG total times based on different shoes, chairs, and locations used by the participants is visualized in Figure 2.

### 3.2. vTUG Timing Data

**Figure 3:**
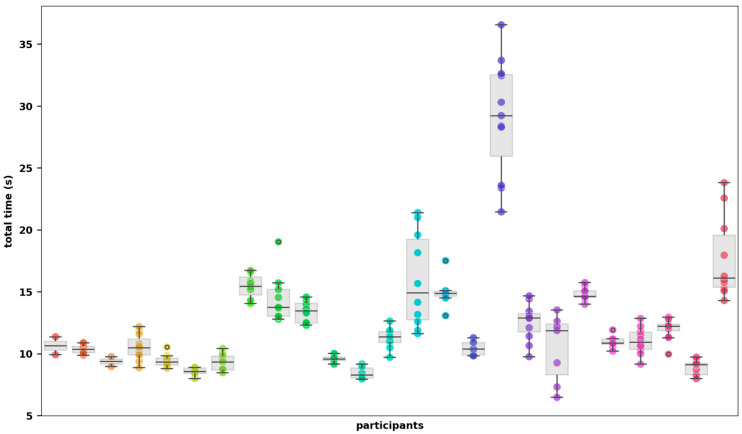
vTUG total time displayed for all participants. Different colours represent individual participants.

The average time required to complete a vTUG was 12.5 s (SD 4.7 s). Figure 3 illustrates the range of total times across all patients and the number of vTUGs recorded. The patient with the longest average time during the study period took 27.6 s on average and also recorded the longest individual vTUG time of 38.7 s. In contrast, the patient with the fastest average time completed the vTUG in 8.6 s, with the single fastest time being 6.2 s. Table 2 provides an overview of the timings for each individual vTUG segment; “time to walk up” was the longest segment.

### 3.3. Relevance of vTUG Stages on Total Time

The time to walk forward showed the strongest correlation with the total time (r = 0.91), while the times to turn 180 degrees and to turn and sit were also strongly correlated (both at r = 0.89). These three segments of the vTUG were confirmed as the most important predictors in the random forest regression.

**Figure 4:**
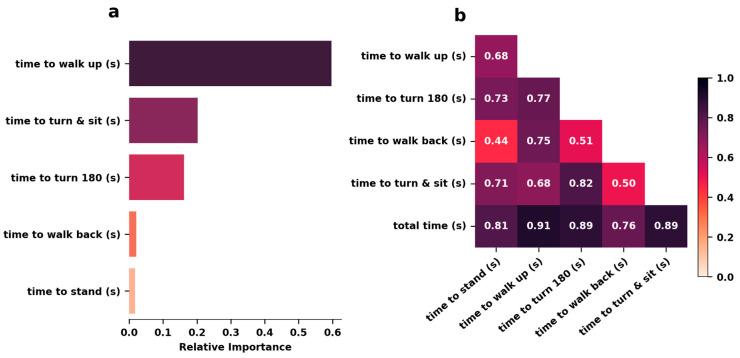
Time to walk up has the highest impact on vTUG total time. (**a**) Relative importance of vTUG segments for predicting vTUG total time. (**b**) Pearson correlations of vTUG segments and vTUG total time.

We performed an 80/20 train-test split to assess the accuracy of the trained model. A grid search for optimal hyperparameters suggested setting the minimum samples per leaf to 1 and using 100 trees in the forest. With these hyperparameters, the random forest was able to predict the vTUG total time with a mean absolute error (MAE) of 0.31 and a mean squared error (MSE) of 0.36. Figure 4 displays both the feature importance plot and the Pearson correlation matrix.

### 3.4. Relationship Between vTUG and MDS UPDRS III

**Figure 5:**
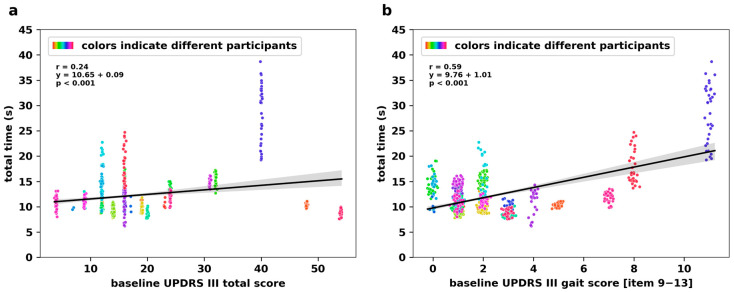
Correlation with MDS UPDRS III gait score indicates vTUG’s ability to detect gait impairments in people with PD. Regression plots for vTUG total time against baseline MDS UPDRS III total score (**a**) and gait subscore derived from the sum of UPDRS III items 9–13 (**b**).

The vTUG total time showed a weak correlation with the MDS UPDRS III total score (r = 0.24; Figure 5a). However, correlation with the derived MDS UPDRS III gait subscore was moderate (r = 0.59; Figure 5b). Linear regression yielded the following equation: y = 10.83 + 0.09x for the MDS UPDRS III total score and y = 9.79 + 1.03 for the MDS UPDRS III gait subscore.

### 3.5. Variance

**Figure 6:**
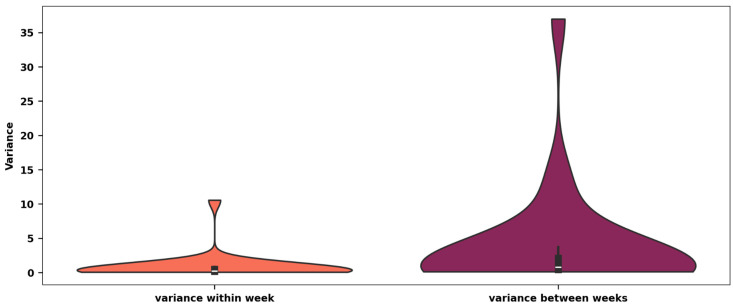
Significant variability emerges in vTUG performance over time, with between-week variance far exceeding within-week consistency. Variances within a single week for three consecutive vTUG tests and variances between the weekly means of vTUG tests over the 12-week study period are depicted in s^2^.

The variance of the total time within the three vTUGs performed consecutively in one week was on average 0.87 s^2^ (SD = 3.19), ranging from 0.01 to 38.6 s^2^. In contrast, the variance of the weekly vTUG means over the course of the 12 weeks of the study was on average 12.55 s^2^ (SD = 4.66), ranging from 6.47 to 36.55 s^2^ (Figure 6). To assess whether there were significant differences between these variances, an F-test was conducted, yielding an F-statistic of 6.50 (*p* < 0.001) and indicating that the variance between weeks was significantly larger than the variance within one measuring time point.

Recognizing that variance is highly sensitive to outliers, we performed a secondary analysis by excluding vTUGs with total times exceeding 20 s, which accounted for only 20 vTUGs. In this filtered dataset, the mean variance within a week was 0.38 s^2^ (SD = 0.61), ranging from 0.01 to 3.81 s^2^, which is still significantly shorter than the mean variance between weeks of 11.74 s^2^ (SD = 2.90) (F-statistic of 34.61; *p* < 0.001).

### 3.6. Influences on vTUG Total Time

Among the tested variables, the UPDRS III gait subscore, patient-reported motion status, shoe type, and chair used significantly improved the AIC, reducing it from 998.19 in the base model to 938.57 in the full model, and were highly significant in the likelihood ratio test (LRT) (*p* < 0.05). In contrast, the UPDRS III total score, age, disease duration, time since last medication, and filming location did not significantly improve the model.

Interestingly, the BIC increased from 1008.43 in the base model to 1036.85 in the full model. This increase may be attributed to BIC’s penalization of models with a high number of parameters, especially those involving categorical variables with multiple levels, such as shoe type and chair used.

Notably, adding chair used to the model led to the UPDRS III gait subscore no longer being significant (*p* = 0.06). Excluding the UPDRS III gait subscore from the final model affected the fit indices differently: the AIC decreased slightly to 939.78, while the BIC decreased to 1034.68. The LRT for the full model was just not significant (*p* = 0.07). Given the mixed changes in the fit indices and the p-value being close to significance, we decided to retain the UPDRS III gait subscore in the full model, which is summarized in Table 3.

### 3.7. End-of-Study Questionnaires

**Figure 7:**
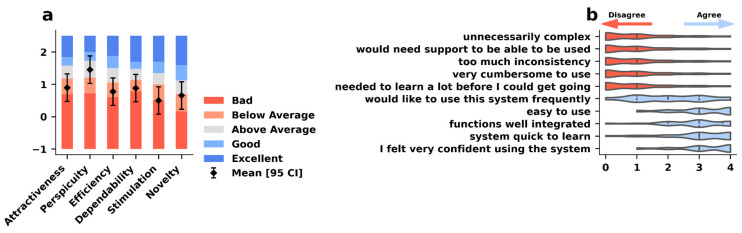
Usability testing revealed above-average perspicuity, emphasizing the ease of learning how to conduct the vTUG, but also indicated some room for improvement in user satisfaction and design. (**a**) Overall UEQ ratings for the vTUG (black diamonds and whiskers representing mean and standard deviation) across the six established domains compared to a benchmark data set [24] (coloured bars). (**b**) Ratings for the 10 SUS items on a 5 item Likert scale (strongly disagree, disagree, neutral, agree, strongly agree).

Twenty-one participants completed the end-of-study questionnaire, results are shown in Figure 7. The mean SUS score was 75,5 (SD = 15.04). The UEQ results indicate that perspicuity received the highest mean score of 1.45 (SD = 1.14), followed by attractiveness with a mean of 0.9 (SD = 0.99). Stimulation had the lowest ranking with a mean score of 0.5 (SD = 0.97). Compared to previous studies [28], perspicuity scored above average, whereas the others scored below average.

## 4. Discussion

This pilot feasibility study was completed by the majority of patients over the period of three months. Despite some errors, a consistently high number of evaluable vTUGs were uploaded, demonstrating the patients’ ability to use the system. This data allowed identification of factors that influence the vTUG performance and should be recorded or standardized in future work.

### 4.1. Video Data

In a direct comparison between different approaches to remotely assess symptoms in PwPD, video-based assessments were generally better accepted than sensor-based assessments and were described by patients to be integrated more easily into their daily lives [28]. Additionally, the vTUG can be completed with minimal time and without additional equipment (except for the stand). This indicates that the method could be employed for research, as an instrument to measure symptom evolution and to detect effects of therapeutic interventions.

The measured times demonstrate significant variance in vTUG performance from one week to the next. This variance likely also arises in measurements performed in clinic, but it is not reported when measurements are carried out at a single time point. Home recordings facilitate repeated measurements and can potentially compensate for such variance and improve the accuracy of measuring symptom severity and disease progression.

In the present study, assessments were performed once per week, and each vTUG consisted of three repetitions. We did not observe significant time variations in those consecutively recorded repetitions, suggesting that they could be reduced to one repetition in PwPD to reduce patient burden and thereby potentially increase adherence to the study protocol. Consistently, the TUG has demonstrated good test-retest reliability [29], whereas inter-session reliability decreases with increasing delay between longitudinal assessments [30].

In some patients, we observed time differences of up to 10 s from week to week, consistent with the previous finding that reliability can be increased by averaging performance of three trials [31]. Some patients, however, showed relatively stable times throughout the study. The simplicity of the vTUG task and app-supported recordings could allow for shorter intervals between recordings in future trials to help differentiate between psychometric properties of the TUG and real changes in disease severity.

### 4.2. Construct Validity

The modest correlation between vTUG total time and UPDRS III total score (r = 0.24) indicates that the vTUG is not suitable to fully capture the entire spectrum of motor impairment in PD. Yet, the vTUG demonstrated moderate associations with specific gait-related items (r = 0.59), suggesting particular utility in assessing gait and balance impairment, which is relevant to the risk of falls.

The vTUG might therefore be applied to further diseases, including Lambert-Eaton-Myasthenia [32] and Essential Tremor [33], or geriatric patients in general. In fact, the TUG was initially developed for the purpose of assessing the risk of falls and functional gait disorders in geriatric patients [34] and the time required to complete the TUG can identify individuals at risk of falling using a threshold of 11.5 s [35].

### 4.3. Questionnaires

Our SUS score of 75.5 is above the mean SUS score for interfaces of 68.5 [36]. The SUS can provide valid scores even with smaller sample sizes [37], so we consider this finding meaningful, consistent with the low attrition rate observed in our cohort.

The UEQ offers some indication of areas where improvements in the assay will have the greatest impact [38]. It exhibited an above-average performance in only one category; the remaining five categories demonstrated performance below the mean. This may initially appear to be an unfavourable outcome. Yet, it is important to note that the questionnaire was not designed exclusively for medical applications. Our objective was to create an app that is as user-friendly as possible for as many patients as possible. It is to be expected that this may result in a reduction in other valuation categories. For example, the weekly video recordings entail additional work without any direct benefit. This could explain the poor result in the stimulation category. We intentionally did not include aspects of gamification in the assay to avoid training effects and hence remain a reliable assay. The high rating in perspicuity indicates that patients did not encounter difficulties in becoming familiar with the app and were able to learn how to use it with ease.

### 4.4. Improvement Suggestions

Home assessments offer the important advantage to assess patients in real-life conditions. We therefore chose to not strictly standardize the recording environment. This approach revealed that the vTUG performance was influenced by various factors such as the chair, footwear, and location, as described previously [39]. In future studies with home assessments, these factors should be standardized—or at least documented. An example of an improvement of the study app could be the option to take a photo of their setup during their first session and store it as a reminder. This would allow patients to remember which items were used during the initial attempt. In addition, the number of consecutively recorded vTUG repetitions could be reduced to one assessment in order to further reduce the burden to the patients and increase adherence to the study protocol. We also noticed that regular interaction with the study team are needed to make sure patients are both performing and recording the vTUG correctly.

### 4.5. Limitations

This study had several limitations. The majority of patients had a less severe form of PD, indicated by a Hoehn and Yahr stage of 1 or 2. The number of patients with a higher stage of PD is not representative, and it is likely that they have more difficulty navigating through the app and following the task without additional support of a spouse or carer. We note, however, that disease-modifying treatments currently enrol patients in early disease stages.

Additionally, the study participants were primarily patients with prior technical experience. We assume that participants who did not feel comfortable using smartphones—or lacked relatives who did so—were less willing to participate in the study. This is expected to be different in therapeutic trials, which may lead to more problems than in our cohort. At the same time, the number of patients with technical experience is expected to grow rapidly with the growing use of technical devices in aging people [40], supported by the ease of use (Figure 7).

Another limitation is the limited clinical characterization of our patient cohort, which did not include the clinical Parkinson subtype and the Levodopa Equivalent Daily Dose (LEDD), both of which could influence variability of gait performance. Different PD subtypes may exhibit distinct motor profiles, which may potentially affect vTUG performance and patient adherence to technology-based assessments.

## 5. Conclusions

The vTUG represents a promising approach to obtaining regular data on symptom severity and fluctuations in the home environment. It is a time-saving and simple method for patients to regularly transmit data to their doctor and has the potential to reduce visit burden for future clinical trials. However, when implementing this approach, it will be essential to pay attention to standardization with regard to factors such as the set-up and the time interval between the last medication intake.

## Figures and Tables

**Table 1:** Baseline Characteristics.

Baseline Characteristic		
Total Participants	25	
	Mean	SD
MDS-UPDRSIII total	20.5	12.2
MDS-UPDRSIII gait subscore *	2.6	2.6
Number of Weeks Recorded	9.8	2.4
vTUG Rounds per Patient	26.3	7.5
vTUG Triplets per Patient	7.5	2.7
	Median	[Q1–Q3]
age	61.0	[58.0–66.0]
age at disease onset	55.0	[51.0–59.0]

* Sum of MDS-UPDRS III Items 9–13.

**Table 2:** Descriptive Statistics for recorded vTUG segments.

vTUG Segment	N	Mean (SD)	Min	Max
time to stand	706	1.29 (0.66)	0.57	8.38
time to walk forward	742	3.43 (1.17)	1.63	10.21
time to turn 180	757	2.1 (0.82)	1.03	7.4
time to walk back	757	2.68 (0.91)	0.87	5.92
time to turn & sit	757	2.81 (1.70)	1.33	15.69
total time	706	12.51 (4.72)	6.17	38.67

All time measures are in seconds.

**Table 3:** Linear mixed effect models evaluating the influence of clinical and environment variables on vTUG total time.

Fixed Effects	Estimates (95 CI)	Std. Error	*p*
Intercept	11.507 (9.921–13.093)	0.809	<0.001
patient-reported motion status (reference category: on)	1.216 (0.487–1.944)	0.372	0.001
UPDRSIII gait	0.381 (−0.018–0.780)	0.204	0.061
Shoe Type (reference category: trainers)			
barefoot	−1.173 (−3.271–0.925)	1.07	0.273
casual	−0.440 (−1.668–0.788)	0.626	0.482
clogs (slip-on)	−0.114 (−3.654–3.425)	1.806	0.949
formal	−0.157 (−2.601–2.287)	1.247	0.9
light trainers	0.077 (−3.747–3.900)	1.951	0.969
sandals	0.900 (−0.730–2.529)	0.831	0.279
sandals (slip-on)	0.740 (−0.698–2.177)	0.733	0.313
slippers	0.441 (−1.720–2.601)	1.102	0.689
slippers (slip-on)	0.692 (−1.047–2.432)	0.888	0.435
socks	0.573(−1.261–2.408)	0.936	0.54
(trainers (slip-on)	1.755 (−1.596–5.106)	1.71	0.305
work boots	0.197 (−2.097–2.491)	1.17	0.866
Chair Type (reference category: dining chair)			
armchair	−2.235 (−3.950–−0.520)	0.875	0.011
camping	−2.600 (−5.455–0.255)	1.457	0.074
clinic	2.073 (−0.526–4.672)	1.326	0.118
director	−2.576 (−5.078–−0.073)	1.277	0.044
function	12.092 (5.922–18.263)	3.148	0.0
garden	−1.406 (−3.143–0.332)	0.887	0.113
hospital	−1.455 (−4.375–1.466)	1.49	0.329
kitchen	−1.460 (−2.794–−0.127)	0.68	0.032
office	0.349 (−2.375–3.073)	1.39	0.802
padded stool	−0.984 (−4.860–2.892)	1.978	0.619
recline	−1.246 (−4.442–1.951)	1.631	0.445
stool	4.565 (2.208–6.923)	1.203	

Data are unstandardized coefficients and (95% confidence intervals).

## Data Availability

Analysis results are available on request from the corresponding author; video material is not available due to ethical restrictions.

## References

[1] Dorsey E.R., Elbaz A., Nichols E., Abbasi N., Abd-Allah F., Abdelalim A., Adsuar J.C., Ansha M.G., Brayne C., Choi J.Y. (2018). Global, regional, and national burden of Parkinson’s disease, 1990–2016: A systematic analysis for the Global Burden of Disease Study 2016. Lancet Neurol..

[2] Wilson D.M., Cookson M.R., Bosch L.V.D., Zetterberg H., Holtzman D.M., Dewachter I. (2023). Hallmarks of neurodegenerative diseases. Cell.

[3] Dinter E., Saridaki T., Diederichs L., Reichmann H., Falkenburger B.H. (2020). Parkinson’s disease and translational research. Transl. Neurodegener..

[4] Alster P., Madetko-Alster N., Otto-Ślusarczyk D., Migda A., Migda B., Struga M., Friedman A. (2023). Role of orexin in pathogenesis of neurodegenerative parkinsonisms. Neurol. Neurochir. Pol..

[5] McGeer P.L., McGeer E.G. (2004). Inflammation and neurodegeneration in Parkinson’s disease. Park. Relat. Disord..

[6] Evers L.J.W., Krijthe J.H., Meinders M.J., Bloem B.R., Heskes T.M. (2019). Measuring Parkinson’s disease over time: The real-world within-subject reliability of the MDS-UPDRS. Mov. Disord..

[7] Lim L., van Wegen E., de Goede C., Jones D., Rochester L., Hetherington V., Nieuwboer A., Willems A., Kwakkel G. (2005). Measuring gait and gait-related activities in Parkinson’s patients own home environment: A reliability, responsiveness and feasibility study. Park. Relat. Disord..

[8] Nieuwboer W.D.W.A. (2001). The Effect of a Home Physiotherapy Program for Persons with Parkinson’s Disease. J. Rehabil. Med..

[9] Nieuwboer A., De Weerdt W., Dom R., Bogaerts K. (2002). Prediction of outcome of physiotherapy in advanced Parkinson’s disease. Clin. Rehabil..

[10] Czech M.D., Badley D., Yang L., Shen J., Crouthamel M., Kangarloo T., Dorsey E.R., Adams J.L., Cosman J.D. (2024). Improved measurement of disease progression in people living with early Parkinson’s disease using digital health technologies. Commun. Med..

[11] Bloem B.R., Dorsey E.R., Okun M.S. (2020). The Coronavirus Disease 2019 Crisis as Catalyst for Telemedicine for Chronic Neurological Disorders. JAMA Neurol..

[12] Ohannessian R., Duong T.A., Odone A. (2020). Global Telemedicine Implementation and Integration Within Health Systems to Fight the COVID-19 Pandemic: A Call to Action. JMIR Public Health Surveill..

[13] Zampieri C., Salarian A., Carlson-Kuhta P., Nutt J.G., Horak F.B. (2011). Assessing mobility at home in people with early Parkinson’s disease using an instrumented Timed Up and Go test. Park. Relat. Disord..

[14] Mathias S., Nayak U., Isaacs B. (1986). Balance in elderly patients: The “get-up and go” test. Arch. Phys. Med. Rehabil..

[15] Podsiadlo D., Richardson S. (1991). The Timed “Up & Go”: A Test of Basic Functional Mobility for Frail Elderly Persons. J. Am. Geriatr. Soc..

[16] Mollinedo I., Cancela J.M. (2020). Evaluation of the psychometric properties and clinical applications of the Timed Up and Go test in Parkinson disease: A systematic review. J. Exerc. Rehabil..

[17] Morris S., Morris M.E., Iansek R. (2001). Reliability of Measurements Obtained with the Timed “Up & Go” Test in People with Parkinson Disease. Phys. Ther..

[18] Lin M., Hwang H., Hu M., Wu H.I., Wang Y., Huang F. (2004). Psychometric Comparisons of the Timed Up and Go, One-Leg Stand, Functional Reach, and Tinetti Balance Measures in Community-Dwelling Older People. J. Am. Geriatr. Soc..

[19] Silva B., Faria C., Santos M., Swarowsky A. (2017). Assessing Timed Up and Go in Parkinson’s disease: Reliability and validity of Timed Up and Go Assessment of biomechanical strategies. J. Rehabil. Med..

[20] Ortega-Bastidas P., Gómez B., Aqueveque P., Luarte-Martínez S., Cano-de-la-Cuerda R. (2023). Instrumented Timed Up and Go Test (iTUG)—More Than Assessing Time to Predict Falls: A Systematic Review. Sensors.

[21] Zampieri C., Salarian A., Carlson-Kuhta P., Aminian K., Nutt J.G., Horak F.B. (2010). The instrumented timed up and go test: Potential outcome measure for disease modifying therapies in Parkinson’s disease. J. Neurol. Neurosurg. Psychiatry.

[22] Postuma R.B., Berg D., Stern M., Poewe W., Olanow C.W., Oertel W., Obeso J., Marek K., Litvan I., Lang A.E. (2015). MDS clinical diagnostic criteria for Parkinson’s disease: MDS-PD Clinical Diagnostic Criteria. Mov. Disord..

[23] Laugwitz B., Held T., Schrepp M., Holzinger A. (2008). Construction and evaluation of a user experience questionnaire. HCI and Usability for Education and Work.

[24] Brooke J. (1986). System Usability Scale (SUS): A Quick-and-Dirty Method of System Evaluation User Information.

[25] Nakagawa S., Schielzeth H. (2013). A general and simple method for obtaining R 2 from generalized linear mixed-effects models. Methods Ecol. Evol..

[26] Sauro J. (2011). A Practical Guide to the System Usability Scale: Background, Benchmarks & Best Practices.

[27] Schrepp M. User Experience Questionnaire Handbook: All You Need to Know to Apply the UEQ Successfully in Your Projects (2015). https://www.ueq-online.org.

[28] Bendig J., Spanz A., Leidig J., Frank A., Stahr M., Reichmann H., Loewenbrück K.F., Falkenburger B.H. (2022). Measuring the Usability of eHealth Solutions for Patients with Parkinson Disease: Observational Study. JMIR Form. Res..

[29] Dal Bello-Haas V., Klassen L., Sheppard M.S., Metcalfe A. (2011). Psychometric Properties of Activity, Self-Efficacy, and Quality-of-Life Measures in Individuals with Parkinson Disease. Physiother. Can..

[30] Luque-Casado A., Novo-Ponte S., Sánchez-Molina J.A., Sevilla-Sánchez M., Santos-García D., Fernández-del-Olmo M. (2021). Test-Retest Reliability of the Timed Up and Go Test in Subjects with Parkinson’s Disease: Implications for Longitudinal Assessments. J. Park. Dis..

[31] Steffen T., Seney M. (2008). Test-Retest Reliability and Minimal Detectable Change on Balance and Ambulation Tests, the 36-Item Short-Form Health Survey, and the Unified Parkinson Disease Rating Scale in People with Parkinsonism. Phys. Ther..

[32] Raja S.M., Sanders D.B., Juel V.C., Harati Y., Smith A.G., Pascuzzi R., Richman D.P., Wu A., Aleš K.L., Jacobus L.R. (2019). Validation of the triple timed up-and-go test in Lambert-Eaton myasthenia. Muscle Nerve.

[33] Monaghan P.G., Murrah W.M., Walker H.C., Neely K.A., Roper J.A. (2024). Evaluating Postural Transition Movement Performance in Individuals with Essential Tremor via the Instrumented Timed Up and Go. Sensors.

[34] World Health Organization (2022). Optimizing Brain Health Across the Life Course: WHO Position Paper.

[35] Nocera J.R., Stegemöller E.L., Malaty I.A., Okun M.S., Marsiske M., Hass C.J. (2013). Using the Timed Up & Go Test in a Clinical Setting to Predict Falling in Parkinson’s Disease. Arch. Phys. Med. Rehabil..

[36] Sauro J., Lewis J.R. (2016). Standardized usability questionnaires. Quantifying the User Experience.

[37] Tullis T.S., Stetson J.N. A Comparison of Questionnaires for Assessing Website Usability. Presented at the UPA 2004 Conference.

[38] Schrepp M., Hinderks A., Thomaschewski J., Marcus A. (2014). Applying the User Experience Questionnaire (UEQ) in Different Evaluation Scenarios. Design, User Experience, and Usability. Theories, Methods, and Tools for Designing the User Experience.

[39] Roussos G., Herrero T.R., Hill D.L., Dowling A.V., Müller M.L.T.M., Evers L.J.W., Burton J., Derungs A., Fisher K., Kilambi K.P. (2022). Identifying and characterising sources of variability in digital outcome measures in Parkinson’s disease. NPJ Digit. Med..

[40] Sixsmith A., Horst B.R., Simeonov D., Mihailidis A. (2022). Older People’s Use of Digital Technology During the COVID-19 Pandemic. Bull. Sci. Technol. Soc..

